# Impacts of age and environment on postnatal microglial activity: Consequences for cognitive function following early life adversity

**DOI:** 10.1371/journal.pone.0306022

**Published:** 2024-06-25

**Authors:** Michaela Fanikos, Skylar A. Kohn, Rebecca Stamato, Heather C. Brenhouse, Kelsea R. Gildawie

**Affiliations:** Department of Psychology, Northeastern University, Boston, Massachusetts, United States of America; Ohio State University, UNITED STATES

## Abstract

Early life adversity (ELA) increases the likelihood of later-life neuropsychiatric disorders and cognitive dysfunction. Importantly, ELA, neuropsychiatric disorders, and cognitive deficits all involve aberrant immune signaling. Microglia are the primary neuroimmune cells and regulate brain development. Microglia are particularly sensitive to early life insults, which can program their responses to future challenges. ELA in the form of maternal separation (MS) in rats alters later-life microglial morphology and the inflammatory profile of the prefrontal cortex, a region important for cognition. However, the role of microglial responses *during* MS in the development of later cognition is not known. Therefore, here we aimed to determine whether the presence of microglia during MS mediates long-term impacts on adult working memory. Clodronate liposomes were used to transiently deplete microglia from the brain, while empty liposomes were used as a control. We hypothesized that if microglia mediate the long-term impacts of ELA on working memory in adulthood, then depleting microglia during MS would prevent these deficits. Importantly, microglial function shifts throughout the neonatal period, so an exploratory investigation assessed whether depletion during the early versus late neonatal period had different effects on adult working memory. Surprisingly, empty liposome treatment during the early, but not late, postnatal period induced microglial activity changes that compounded with MS to impair working memory in females. In contrast, microglial depletion later in infancy impaired later life working memory in females, suggesting that microglial function during late infancy plays an important role in the development of cognitive function. Together, these findings suggest that microglia shift their sensitivity to early life insults across development. Our findings also highlight the potential for MS to impact some developmental processes only when compounded with additional neuroimmune challenges in a sex-dependent manner.

## 1. Introduction

Childhood maltreatment increases the likelihood of later-life neuropsychiatric disorders, such as anxiety, schizophrenia, and substance use disorders [1–3]. Cognitive dysfunction is a central feature of these disorders [4–10]. Additionally, early life adversity (ELA) affects brain regions important for cognition, such as the prefrontal cortex (PFC) and hippocampus, which in turn influences cognitive development [11–16]. Moreover, inflammation is strongly linked to both neuropsychiatric disorders and cognitive impairment [17–21].

Microglia—the resident immune cell of the brain—have a variety of functions, including releasing cytokines, refining synapses, and phagocytosing debris and dead cells [22]. These cells are critical for normative brain development [22] shaping synaptic connections [23,24] and driving sexually dimorphic behavioral maturation [25]. Importantly, microglial morphology and function are age-dependent and change over the neonatal period [26,27]. For example, in the first weeks of life, microglia shift from being amoeboid to having a more ramified structure [28,29]. Moreover, microglia regulate the density of synapses as the brain develops; for example, in rodents postnatal day (P)8-P10 is a sensitive window for microglial engulfment of thalamocortical synapses [30]. Because of these shifts in microglial properties in the postnatal period, the way microglia respond to adverse events may depend on the timing of these insults.

Microglia colonize the brain early in embryogenesis and are long-lived cells [31–33]. The perinatal period may therefore be a sensitive period for long-term changes in microglial function and related cognitive outcomes. For example, neonatal, but not juvenile, *Escherichia coli* infection conferred memory deficits following later-life immune stimulation with lipopolysaccharide [34]. The memory deficits observed in neonatally infected rats were driven by heightened IL-1b, suggesting a critical role for enhanced microglial neuroinflammatory signaling after early-life infection [35,36].

In addition to immune challenge, stress can also impact microglia via changing microglial density, morphology, and cytokine release [37–41]. In rodents, maternal separation (MS) is a common model of ELA that changes immune properties of the brain. MS increases the number of amoeboid microglia [42] and increases cytokine expression in the PFC [43] during the preweaning period. Additionally, our lab has shown that MS has enduring effects on the PFC, with juvenile rats exhibiting larger microglia soma size [44] and adolescent male rats displaying higher expression of tumor necrosis factor, a proinflammatory cytokine [45]. Indeed, MS worsens performance on the PFC-dependent win-shift task in both early and late adolescence [46,47]. However, it is unclear if the effects of MS on microglia and adolescent working memory are directly related; likewise, it is unknown if these impairments in working memory persist through adulthood.

Experimental manipulation of microglial activity in rats is notoriously challenging [48], especially during development. Most of the techniques used to manipulate microglia have broad effects. For example, minocycline, a second generation tetracycline antibiotic, has been used to inhibit microglia, but the mechanisms by which this occurs and the extent to which this is true have been debated [49]. Other methods include depleting microglia from the brain using genetic ablation or pharmacologic depletion with colony-stimulating factor receptor 1 (CSF1R) inhibition or clodronate liposomes [50–54]. In the present study, clodronate liposomes were used to transiently deplete microglia from the developing brain. Clodronate liposomes are formulated by packaging the cytotoxic compound clodronate in liposomes, which are used as a drug delivery vehicle to phagocytes, such as microglia. Upon phagocytosis of the clodronate liposome, the cell undergoes apoptosis and is eliminated [55,56]. Empty liposomes, which do not contain the cytotoxic clodronate, are used as a vehicle control. Central clodronate liposome administration in neonates has implicated microglia as critical regulators in the development of social and anxiety-like behaviors in juvenility, adolescence, and adulthood [54,57].

The present study aimed to assess the developmental period within which microglia may play a role in the long-term consequences of ELA. To assess this, microglia were depleted at two different periods during MS. This design was important given that microglial activity changes throughout the preweaning period and there may be a sensitive period for the impact of MS on microglial function. Because clodronate liposomes transiently deplete microglia, we were poised to deplete microglia either during early postnatal life or later in infancy. We hypothesized that if microglia mediate impairments to adult working memory following MS, then depleting microglia during MS will prevent these impairments. An exploratory investigation further tested whether early or late depletion would be more effective in preventing working memory deficits. However, an important consideration for developmental microglial manipulations is how methodological techniques may themselves affect microglial morphology and function. Therefore, we also assessed how administration of empty liposomes may itself alter microglia during infancy.

## 2. Methods

All experiments were conducted in accordance with the 1996 Guide for the Care and Use of Laboratory Animals (NIH) with approval from the Institutional Animal Care and Use Committee at Northeastern University.

### 2.1 Subjects and maternal separation (MS)

All subjects were bred in an in-house colony with Sprague-Dawley rats originally obtained from Charles River Laboratories (Wilmington, MA). Animals were housed in standard polycarbonate wire-top cages and pine shavings bedding in a facility controlled for temperature (22–23°C) and humidity with a 12-hour light/dark cycle (light period 0700–1900). Food (ProLab 5P00) and water (glass bottles) were available ad libitum to dams and weaned subjects. The day of birth was denoted postnatal day (P)0. On P1, litters were culled to 10 pups (five males and five females when possible), and randomly assigned to control (Con) or maternal separation (MS) rearing conditions. Pups in the Con litters were left undisturbed except for brief handling for three to five minutes twice per week and weekly cage changes. From P2 through P20, pups in MS litters were removed from their cage for 4 hours/day. From P2-P10, each pup was isolated in individual plastic cups with home cage bedding in a circulating water bath at 35°C. From P11-20, the age at which pups can thermoregulate, pups were isolated in individual mouse cages with home cage bedding. Rats were weaned on P21 and housed with a same-sex cagemate. To avoid litter effects, no more than two pups per litter were assigned to each experimental group. Therefore, animals were randomly selected from 48 Con litters and 36 MS litters. From individual litters, 1–2 pups per sex per condition were used for each experiment, with remaining pups allocated to other ongoing studies.

### 2.2 Experimental design

#### 2.2.1 Experiment 1

**Fig 1:**
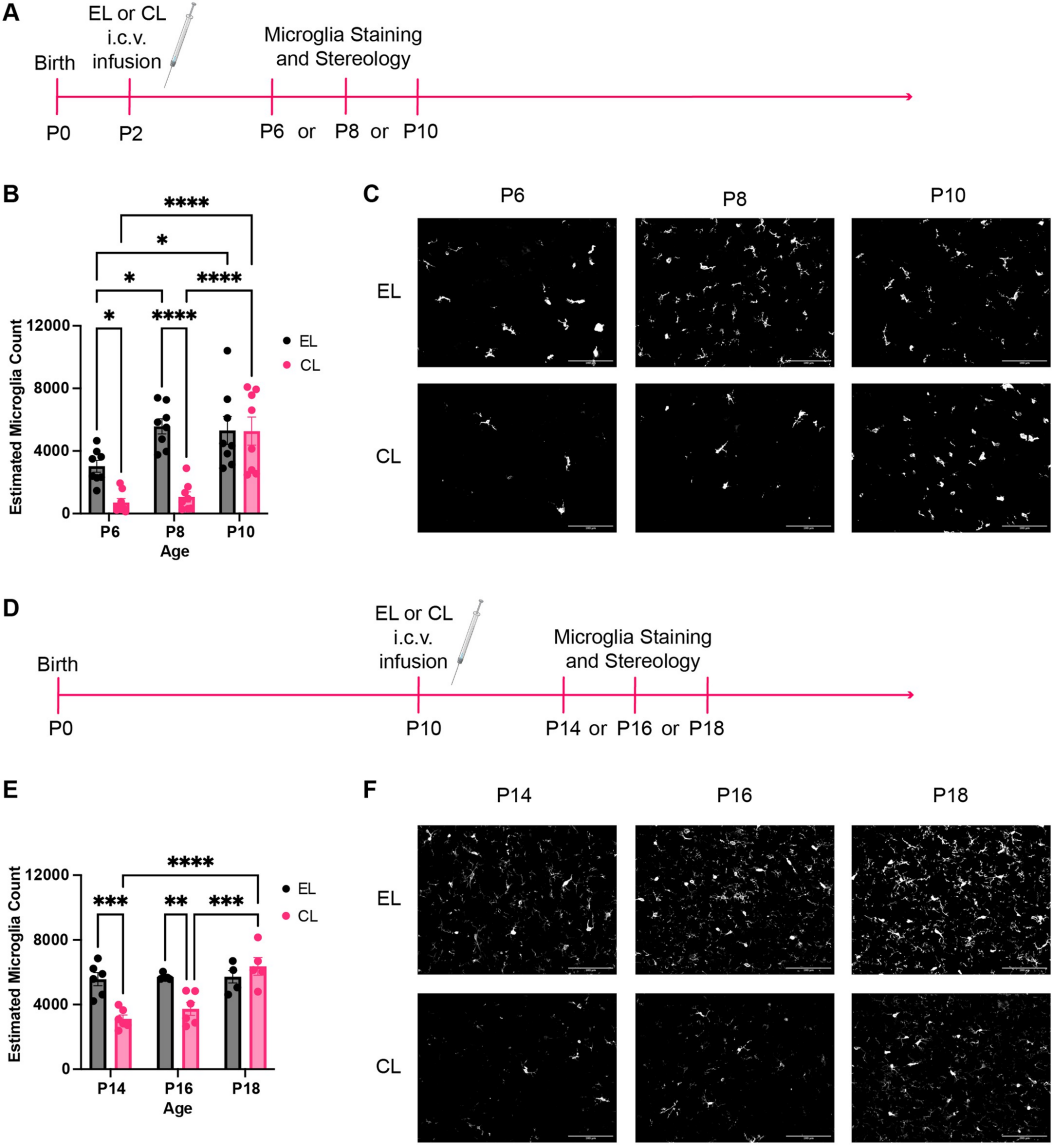
Experiment 1: Microglial repopulation following clodronate liposome administration. A) Experimental timeline for P2 microglia depletion. Following infusion of empty liposomes (EL) or clodronate liposomes (CL) on P2, pups were sacrificed on P6, P8, or P10 for Iba1 staining and stereological counting. B) Estimated microglia count following P2 CL or EL infusion; n = 8. C) Representative photomicrographs of Iba1 stained slices from P2 EL and CL-treated animals sacrificed on P6, P8, or P10. Scale bar represents 100 μm. D) Experimental timeline for P10 microglia depletion. Following infusion of EL or CL on P10, pups were sacrificed on P14, P16, or P18 for Iba1 staining and stereological counting. E) Estimated microglia count following P10 CL or EL infusion; n = 4–6. F) Representative photomicrographs of Iba1 stained slices from P10 EL and CL-treated animals sacrificed on P14, P16, or P18. Scale bar represents 100 μm. *p<0.05, **p<0.01, ***p<0.001, ****p<0.0001. Created with BioRender.com.

Experiment 1 aimed to confirm that clodronate liposomes deplete microglia following P2 or P10 intracerebroventricular (i.c.v.) infusion. Pups were sacrificed 4, 6, or 8 days following the infusion for Iba1 staining and stereology (Fig 1A and 1D). A total of 48 pups received the infusion on P2, with 8 pups in each group. A total of 33 pups received the infusion on P10, with 4–6 pups in each group. All groups contained both Con and MS pups of both sexes.

#### 2.2.2 Experiment 2

**Fig 2:**
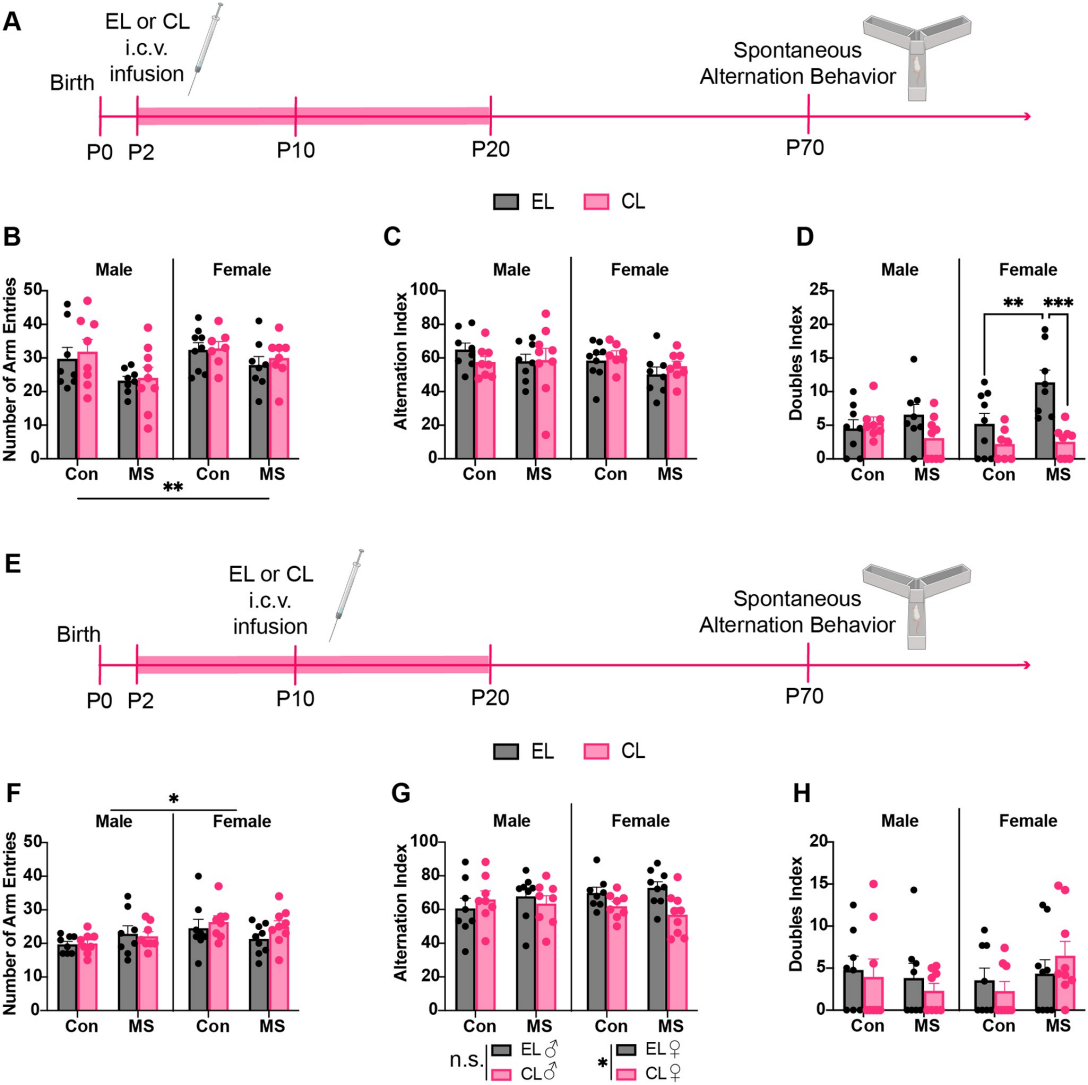
Experiment 2: Effects of maternal separation and microglial depletion on spontaneous alternation test of working memory. A) Experimental timeline for P2 injected cohort. Pups received empty liposome (EL) or clodronate liposome (CL) infusion on P2 and underwent maternal separation or control rearing from P2 to P20, indicated by pink highlighted region on timeline. After weaning on P21 rats were left undisturbed until spontaneous alternation testing on P70. B) P2 cohort total arm entries in spontaneous alternation test; n = 7–9. C) P2 cohort alternation index; n = 7–9. D) P2 cohort doubles index; n = 7–9. E) Experimental timeline for P10 injected cohort. Pups underwent maternal separation or control rearing from P2 to P20, indicated by pink highlighted region on timeline. On P10 pups received EL or CL infusion. After weaning on P21 rats were left undisturbed until spontaneous alternation testing on P70. F) P10 cohort total arm entries in spontaneous alternation test; n = 8–9. G) P10 cohort alternation index; n = 8–9. H) P10 cohort doubles index; n = 8–9. *p<0.05, **p<0.01, ***p<0.001. Created with BioRender.com.

Experiment 2 assessed if microglia play a role in the long-term impacts of MS on working memory in adulthood. Microglia were depleted at two different timepoints during MS in two separate cohorts of animals. In the early timepoint cohort, pups received infusion of clodronate liposomes or empty liposomes on P2; in the late timepoint cohort, pups received infusion of clodronate liposomes or empty liposomes on P10. Both cohorts were either control reared or MS-reared from P2-P20 and weaned on P21. Working memory in the spontaneous alternation test was tested on P70 (Fig 2A and 2E). A total of 65 animals were assessed in the early cohort, with 7–9 animals in each group. A total of 66 animals were assessed in the late cohort, with 8–9 animals in each group.

#### 2.2.3 Experiment 3

**Fig 3:**
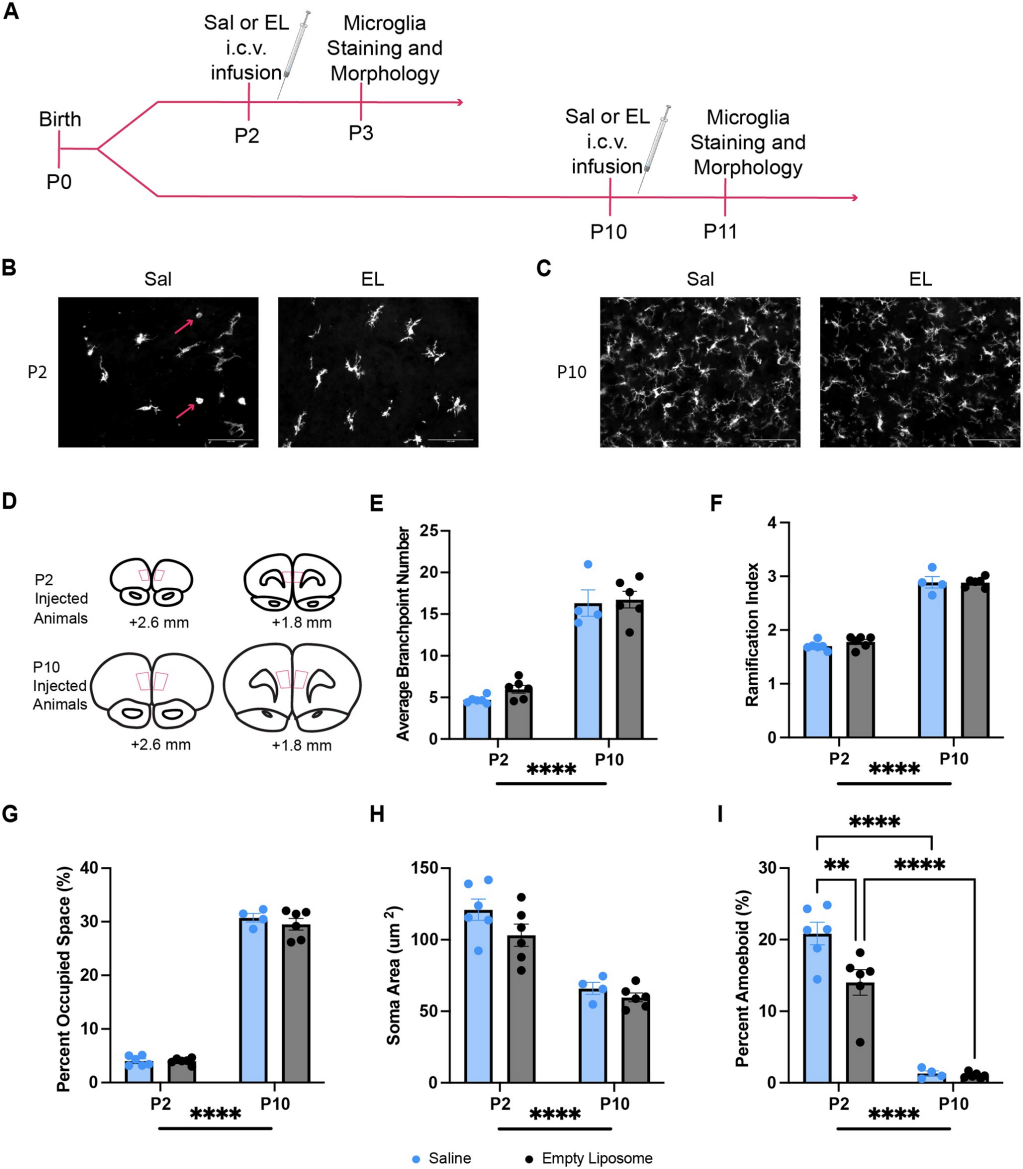
Experiment 3: Age-dependent effects of liposome treatment on microglia morphology. A) Experimental timeline to assess age-dependent effects of liposome treatment. Pups received Saline (Sal) or empty liposome (EL) infusion on P2 or P10. 24 hours later pups were sacrificed for Iba1 staining and morphology analysis. B) Representative photomicrographs for P2 injected Sal and EL treated pups. Arrows indicate representative amoeboid microglia. Scale bar represents 100 μm C) Representative photomicrographs for P10 injected Sal and EL treated pups. Scale bar represents 100 μm. D) Sections adapted from neonatal rat brain atlas [58] illustrating where images were taken for morphology analyses. E) Average number of branchpoints; n = 4–6. F) Ramification index; n = 4–6. G) Percent of space occupied by microglia; n = 4–6. H) Soma area; n = 4–6. I) Percent amoeboid microglia; n = 4–6. **p<0.01, ****p<0.0001. Created with BioRender.com.

Experiment 3 investigated if liposome treatment itself changes microglia morphology in an age dependent manner. Pups received empty liposome or saline infusion on P2 or P10 and were sacrificed 24 hours later for Iba1 staining and morphological analysis (Fig 3A). A total of 22 pups were used, with 4–6 pups in each group. Each group contained both Con and MS pups of both sexes.

### 2.3 Microglial depletion procedure

For the early depletion study, MS and Con subjects were cryoanesthetized for bilateral i.c.v. infusions of undiluted clodronate liposomes (Clodrosome^®^) or empty liposomes (Encapsome^®^) on P2. For each hemisphere, 1 μL of clodronate liposomes or empty liposomes were injected slowly with a 25-gauge 5-μL Hamilton syringe 1 mm caudal to bregma and 1 mm lateral to the midline. Additionally, a small amount of Evan’s Blue Dye (E2129, Sigma-Aldrich) was injected with the clodronate liposomes or empty liposomes, to ensure proper placement in the lateral ventricles. Cryoanesthesia and depletion procedures lasted 10–12 min/pup. Following injections, pups were placed in a mouse cage atop a heating pad until ready to be returned to the dam.

For the late depletion study, Con and MS subjects were anesthetized with isoflurane via inhalation (1–4% mg/kg) for the duration of the i.c.v. injection. Local anesthetic, lidocaine, was administered to the skin prior to incision. At P10 the skull is too thick to directly insert a needle through the skull, so the skulls were drilled at the incision site prior to placement. For each hemisphere, 1 μL of clodronate liposomes or empty liposomes were injected slowly with a 25-gauge 5-μL Hamilton syringe 1 mm caudal to bregma and 1 mm lateral to the midline. Additionally, a small amount of Evan’s Blue Dye was injected with the clodronate liposomes or empty liposomes, to ensure proper placement in the lateral ventricles. Anesthesia and depletion procedures lasted 10–12 min/pup. Following injections, pups were placed in a mouse cage atop a heating pad until ready to be returned to the dam.

### 2.4 Microglial depletion confirmation

To confirm and characterize the time-course of microglial depletion following P2 clodronate liposome administration, P6, P8, and P10 pups were deeply cryoanesthetized and transcardially perfused with ice cold 0.9% saline followed by 4% paraformaldehyde (PFA) made from 0.1 M phosphate buffered saline (PBS). To confirm and characterize the time-course of the microglial depletion following P10 clodronate liposome administration, P14, P16, and P18 pups were deeply anesthetized with carbon dioxide and transcardially perfused with ice cold 0.9% saline followed by 4% PFA made from 0.1M PBS. Brains were removed and placed in 4% PFA for three days. Tissue was then cryoprotected in 30% sucrose and sectioned directly onto slides with a cryostat set to 10 μm and stored at -80°C until immunohistochemical staining. To label Iba1+ microglia in the frontal lobe, slides containing sections were first washed in PBS. Sections were then blocked in 10% normal donkey serum (017-000-121, Jackson ImmunoResearch, West Grove, PA) and incubated for 1 hour at room temperature. Sections were then incubated for 1 hour at room temperature in primary rabbit polyclonal anti-ionized calcium binding adaptor molecule (Iba1; 019–19741, 1:1000, Wako, Richmond, VA). Sections were then incubated in Alexa Fluor 594 donkey anti-rabbit secondary fluorescent antibody (1:500, A21207, Invitrogen, Eugene, OR) for 1 hour at room temperature. Sections were washed between steps in 0.1 M PBS containing 0.2% Triton X-100 (Fisher Scientific). Lastly, sections were dipped in xylenes and cover-slipped with ProLong Gold antifade mounting reagent (P36930, Invitrogen). All immunohistochemical batches included subjects from each experimental condition to obviate batch-specific variances in staining intensity.

### 2.5 Microglia depletion quantification

The Iba1 stained slices were imaged using a Zeiss Axio Imager M2 and stereology was performed using the Stereo Investigator Optical Fractionator Probe (MBF Bioscience). For stereology performed on P6, P8, and P10, three sections were selected at random between bregma 2.8 mm and bregma 1.8 mm. For stereology performed on P14, P16, and P18, three sections were selected at random between bregma 2.0 mm and bregma 3.0 mm. Regions were outlined using a developmental rat brain atlas as a guide [58] with magnification set to 2.5x. While neonates have not yet developed PFC cytoarchitecture, we used ROIs within the frontal cortex to assess microglial depletion in an area that was distant from the periventricular area immediately surrounding the injections. Additionally, we have previously observed neuroinflammatory changes following ELA in the mPFC, indicating this is a relevant region for microglial impacts during ELA [44,45]. Counting was performed by a trained experimenter masked to condition, with magnification set to 20x and exposure time kept constant at 100 ms.

### 2.6 Spontaneous alternation

In both the P2 injected cohort and the P10 injected cohort, behavioral testing was performed on P70. The spontaneous alternation test was used as a measure of working memory [59]. The test was performed using three arms in a “Y” configuration of an eight-arm radial maze in dim lighting. The rat was placed in the “A” arm of the maze, facing away from the center, and was allowed to freely explore for an eight-minute video-recorded trial. A gray curtain was placed around the maze to prevent the use of distal cues to aid the animals in the task, which constrains the animals to working memory to determine which arms they had just visited. The maze was cleaned with 50% ethanol between subjects. The order of arm entries was reported by a trained experimenter masked to animal condition. The total number of arm entries, total alternations, and total doubles were calculated. Consecutive entries into each of the three arms was considered an alternation and a re-entry into the same arm was considered a double. The alternation index and doubles index were calculated with the following equations.


$$alternation\;index=\;\frac{number\;of\;alternations}{total\;arm\;entries-2}$$

$$doubles\;index=\;\frac{number\;of\;doubles}{total\;arm\;entries-1}$$

### 2.7 Microglial morphology analysis following liposome treatment

To assess if liposome treatment alone alters microglia morphology in an age-dependent manner, pups received i.c.v. infusion of either saline or empty liposomes on P2 or P10 and were sacrificed 24 hours later for brain collection. The infusion and perfusion protocols were the same as described in sections 2.2 and 2.3, respectively. Briefly, P2 pups were cryoanesthetized and received 1 μL bilateral i.c.v. infusion of saline or empty liposomes. Following injections, pups were placed in a mouse cage atop a heating pad until ready to be returned to the dam. Twenty-four hours later, on P3, pups were deeply cryoanesthetized and transcardially perfused with ice cold 0.9% saline followed by 4% PFA made from 0.1 M PBS. P10 pups were anesthetized with isoflurane via inhalation (1–4% mg/kg) and received 1 μL bilateral i.c.v. infusion of saline or empty liposomes. A local anesthetic, lidocaine, was administered to the skin prior to incision. Following injections, pups were placed in a mouse cage atop a heating pad until ready to be returned to the dam. Twenty-four hours later, on P11, pups were deeply anesthetized with carbon dioxide and transcardially perfused with ice cold 0.9% saline followed by 4% PFA made from 0.1 M PBS. Following perfusions for both age groups, brains were extracted and post-fixed in 4% PFA for 24 hours before being cryoprotected in 30% sucrose. Frontal cortices were sectioned onto slides with a cryostat set to 40 μm and stored at -80°C until immunohistochemical staining. Iba1+ microglia were labeled as described in section 2.4 above, except incubation times were extended to 1.5 hours to ensure penetration of antibodies throughout the 40 μm tissue.

### 2.8 Microglial morphology quantification

The Iba1 stained frontal cortices were imaged using a Zeiss Axio Imager M2. Seven slices between bregma 2.6 and 1.8 mm were used, with one z-stack taken from each hemisphere, for a total of 14 z-stacks for each subject. The top and bottom of each slice were set and 0.5 μm z-stacks were taken at 20x magnification (442 μm x 331 μm). The exposure time was set to 100 ms for all z-stacks.

3DMorph, a semi-automatic MATLAB-based program was used to assess morphological characteristics of each microglia, including ramification index, percent coverage, and branch points [60]. An average of these indices for all microglia per subject was calculated. Based on the 3DMorph output, the percent of microglia without branches, designated amoeboid microglia for simplicity, was calculated for each subject. In ImageJ, the soma size of each microglia was measured using the “Freehand line” followed by the “Analyze and Measure” function [61]. Somas were drawn by a trained experimenter masked to the animal condition. The average soma area for all microglia per subject was then calculated.

### 2.9 Statistical analysis

Statistical analyses were performed using GraphPad Prism 10 (GraphPad Software Inc, La Jolla, CA, USA). Group differences for the microglial depletion confirmation studies were analyzed using 2-way ANOVAs with a treatment factor (empty liposomes and clodronate liposomes) and age factor (P6, P8, and P10 for P2 treatment or P14, P16, and P18 for P10 treatment). The two depletion timepoints, P2 or P10, were analyzed separately. Group differences for behavioral testing were analyzed first with 3-way ANOVAs with a rearing factor (Con and MS), Treatment factor (empty liposomes and clodronate liposomes), and sex factor (Males and Females). Group differences for microglia morphology analyses were analyzed using 2-way ANOVAs with a treatment factor (saline and empty liposomes) and age factor (P2 and P10). Before analysis, data were assessed for outliers using Grubbs’ Test. Normality of data distribution was analyzed for all variables using the Shapiro-Wilk Test of Normality. In all analyses, relevant interactions were followed up with Holm-Sidak multiple comparison tests. All data are visually presented as group mean ± standard error of the mean (SEM) with individual subjects represented with dots, and statistical significance was considered as p <0.05. In the absence of significant interactions, significant main effects are displayed on all graphs. When both significant main effects and interactions were present, main effects are only displayed if both relevant post-hoc comparisons were significant, otherwise only the pairwise comparisons are displayed. Effect sizes are reported as partial eta-squared for significant main effects and interactions, and as Cohen’s d for significant Holm-Sidak multiple comparison tests.

## 3. Results

### 3.1 Microglial repopulation

The effect of P2 i.c.v. clodronate liposome infusion on microglia count was assessed at P6, P8, and P10. A 2-way ANOVA of estimated microglia count showed main effects of age (F_2,42_ = 16.55, P<0.0001, η_p_^2^ = 0.441) and of treatment (F_1, 42_ = 22.03, P<0.0001, η_p_^2^ = 0.344), but also a significant interaction effect between age and treatment (F_2,42_ = 6.976, P = 0.0024, η_p_^2^ = 0.249; Fig 1A–1C). Holm-Sidak’s multiple comparisons tests showed a significant difference between empty liposome and clodronate liposome-treated animals on P6 (t_42_ = 2.745, P = 0.0436, d_Cohen_ = 2.577) and on P8 (t_42_ = 5.333 P<0.0001, d_Cohen_ = 3.882), but not on P10 (t_42_ = 0.05107, P = 0.9639). There were also significant differences in the number of microglia in clodronate liposome-treated animals between P6 and P10 (T_42_ = 5.40, P<0.0001, d_cohen_ = 2.46) and between P8 and P10 (T_42_ = 4.974, P<0.0001, d_cohen_ = 2.21), indicating that microglial depletion with clodronate liposomes is transient. Additionally, we observed a developmental increase in the number of microglia in empty liposome-treated animals, such that there were significantly more microglia on P8 compared to P6 (T_42_ = 3.108, P = 0.0256, d_cohen_ = 2.075) and on P10 compared to P6 (T_42_ = 2.706, P = 0.0436, d_cohen_ = 1.176) (Fig 1B). Together these data indicate that clodronate liposome significantly reduces microglia count following administration for approximately eight days, which coincides with a developmental time period when microglia count is normally increasing.

The effect of P10 i.c.v. clodronate liposome infusion on microglia count was assessed at P14, P16, and P18. A 2-way ANOVA of estimated microglia count showed main effects of age (F_2, 26_ = 9.355, P = 0.0009, η_p_^2^ = 0.418) and of treatment (F_1,26_ = 15.01, P = 0.0006, η_p_^2^ = 0.366) but also a significant interaction between age and treatment (F_2,26_ = 9.173, P = 0.0010, η_p_^2^ = 0.414; Fig 1C–1E). Holm-Sidak’s multiple comparisons test shows a significant difference between empty liposome and clodronate liposome-treated animals on P14 (t_26_ = 4.802, P = 0.0004, d_Cohen_ = 2.984) and on P16 (t_27_ = 3.664, P = 0.0067, d_Cohen_ = 2.653), but not P18 (t_26_ = 1.246, p = 0.7184). Additionally, there were significantly more microglia on P18 compared to P14 (t_26_ = 6.060, P<0.0001, d_Cohen_ = 3.497) and on P18 compared to P16 (t_26_ = 4.890, P = 0.0004, d_Cohen_ = 2.400), again indicating that microglial depletion with clodronate liposomes is transient. There were no developmental differences in microglia count in empty liposome-treated animals (Fig 1E). For full statistical results see Table 1. Overall, these data suggest that microglia are depleted for approximately eight days following both P2 and P10 clodronate liposomes administration.

**Table 1:** Microglial repopulation statistical results.

**P2 Administration Microglia Count**	**Two-Way ANOVA**			
	**Comparison**	**Test Statistic**	**P**	**η**_**p**_^**2**^
	Treatment	F_1,42_ = 22.03	P<0.0001	η_p_^2^ = 0.344
	Age	F_2,42_ = 16.55	P<0.0001	η_p_^2^ = 0.441
	Treatment x Age	F_2,42_ = 6.976	P = 0.0024	η_p_^2^ = 0.249
	**Relevant Post Hoc Tests**			
	**Comparison**	**Test Statistic**	**P**	**Cohen’s d**
	P6: EL vs CL	T_42_ = 2.745	P = 0.0436	d = 2.577
	P8: EL vs CL	T_42_ = 5.333	P<0.0001	d = 3.882
	P10: EL vs CL	T_42_ = 0.051	P = 0.9639	d = 0.017
	CL P6 vs P8	T_42_ = 0.430	P = 0.9639	d = 0.451
	CL: P6 vs P10	T_42_ = 5.400	P<0.0001	d = 2.460
	CL: P8 vs P10	T_42_ = 4.970	P<0.0001	d = 2.210
	EL: P6 vs P8	T_42_ = 3.018	P = 0.0256	d = 2.075
	EL: P6 vs P10	T_42_ = 2.706	P = 0.0436	d = 1.176
	EL: P8 vs P10	T_42_ = 0.312	P = 0.9639	d = 0.129
**P10 Administration Microglia Count**	**Two-Way ANOVA**			
	**Comparison**	**Test Statistic**	**P**	**η**_**p**_^**2**^
	Treatment	F_1,26_ = 15.01	P = 0.0010	η_p_^2^ = 0.366
	Age	F_2,26_ = 9.355	P = 0.0009	η_p_^2^ = 0.419
	Treatment x Age	F_2,26_ = 9.173	P = 0.0006	η_p_^2^ = 0.414
	**Relevant Post Hoc Tests**			
	**Comparison**	**Test Statistic**	**P**	**Cohen’s d**
	P14: EL vs CL	T_26_ = 4.802	P = 0.0004	d = 2.984
	P16: EL vs CL	T_26_ = 3.664	P = 0.0067	d = 2.653
	P18: EL vs CL	T_26_ = 1.246	P = 0.7184	d = 0.665
	CL: P14 vs P16	T_26_ = 1.227	P = 0.7184	d = 0.772
	CL: P14 vs P18	T_26_ = 6.060	P<0.0001	d = 3.497
	CL: P16 vs P18	T_26_ = 4.890	P = 0.0004	d = 2.400
	EL: P14 vs P16	T_26_ = 0.256	P = 0.9920	d = 0.182
	EL: P14 vs P18	T_26_ = 0.095	P = 0.9920	d = 0.055
	EL: P16 vs P18	T_26_ = 0.139	P = 0.9920	d = 0.129

Statistical results for Experiment 1: Microglial Repopulation. Significant findings are shaded gray. Empty liposome (EL), clodronate liposome (CL).

### 3.2 Spontaneous alternation behavior

The spontaneous alternation task on P70 assessed working memory in adulthood after MS or control rearing with either empty liposomes or clodronate liposomes at P2 (Fig 2A–2D) or P10 (Fig 2E–2H). For each outcome measure (total arm entries, doubles index, or alternations index) analyzed with a 3-way ANOVA using sex, rearing, and treatment as factors.

Following the P2 injections, a 3-way ANOVA revealed a significant main effect of rearing on total arm entries (F_1,57_ = 8.084, P = 0.006, η_p_^2^ = 0.124) such that MS-exposed animals made fewer arm entries, regardless of treatment or sex (Fig 2B). A 3-way ANOVA of the alternation index did not reveal any main effects or interactions (Fig 2C). A 3-way ANOVA of the doubles index revealed a main effect of treatment (F_1,57_ = 15.59, P = 0.0002, η_p_^2^ = 0.215) as well as rearing x treatment (F_1,57_ = 7.716, P = 0.007, η_p_^2^ = 0.119) and sex x treatment (F_1,57_ = 6.265, P = 0.015, η_p_^2^ = 0.099) interactions. Follow-up 2-way ANOVAs were performed in each sex separately. While there were no significant main effects or interactions in the male doubles index, there were main effects of rearing (F_1,28_ = 5.445, P = 0.027, η_p_^2^ = 0.162) and treatment (F_1,28_ = 18.14, P = 0.0002, η_p_^2^ = 0.393), as well as a rearing x treatment interaction (F_1,28_ = 4.422, P = 0.045, η_p_^2^ = 0.136) in the female doubles index. Holm-Sidak’s multiple comparisons test of the female doubles index revealed that MS empty liposome-treated females had a significantly higher doubles index compared to Con empty liposome-treated females (t_28_ = 3.241, P = 0.0092, d_Cohen_ = 1.261). Additionally, MS clodronate-treated females had a significantly lower doubles index compared to MS empty liposome-treated females (t_61_ = 4.516, P = 0.0004, d_Cohen_ = 2.215) (Fig 2D). These data indicate that MS impairs working memory in empty liposome-treated females, but depleting microglia at P2 during MS prevents this impairment. Additionally, there were no significant differences in the doubles index between Con females that received empty liposomes versus to clodronate liposomes (t_28_ = 1.519, P = 0.261) or between clodronate liposome-treated females that were MS versus Con reared (t_28_ = 0.158, P = 0.876) indicating that the main effect of treatment on the doubles index was driven by the treatment x rearing interaction.

Following the P10 injections, a 3-way ANOVA of the arm entries revealed a significant main effect of sex (F_1,58_ = 6.127, P = 0.016, η_p_^2^ = 0.100) but no interactions (Fig 2F). A 3-way ANOVA of the alternation index revealed a sex x treatment interaction (F_1,58_ = 4.049, P = 0.049, η_p_^2^ = 0.070) (Fig 2G). Follow-up 2-way ANOVAs were performed in each sex separately. While there were no significant main effects or interactions in the male alternation index there was a main effect of treatment in the female alternations index such that females treated with clodronate liposomes had a lower alternation index than females treated with empty liposomes. No post hoc pairwise comparisons were performed as there was no significant rearing x treatment interaction (F_1,30_ = 11.54, P = 0.002, η_p_^2^ = 0.277) (Fig 2G). A 3-way ANOVA of the doubles index did not reveal any main effects or interactions (Fig 2H). For full statistical results see Table 2. Thus, in this late-liposome administration cohort, MS did not impair working memory, however the absence of microglia in the late-infant brain impaired working memory in females.

**Table 2:** Spontaneous alternation statistical results.

**P2 Cohort**	**Three-Way ANOVA**				
		**Comparison**	**Test Statistic**	**P**	**η**_**p**_^**2**^
	Arm Entries	Rearing	F_1,57_ = 8.084	P = 0.006	η_p_^2^ = 0.124
		Sex	F_1,57_ = 3.46	P = 0.068	η_p_^2^ = 0.057
		Treatment	F_1,57_ = 0.524	P = 0.472	η_p_^2^ = 0.009
		Rearing x Sex	F_1,57_ = 0.803	P = 0.374	η_p_^2^ = 0.014
		Rearing x Treatment	F_1,57_ = 0.003	P = 0.953	η_p_^2^<0.001
		Sex x Treatment	F_1,57_ = 0.003	P = 0.953	η_p_^2^<0.001
		Rearing x Sex x Treatment	F_1,57_ = 0.152	P = 0.698	η_p_^2^ = 0.003
	Alternation Index	Rearing	F_1,57_ = 2.72	P = 0.105	η_p_^2^ = 0.045
		Sex	F_1,57_ = 1.33	P = 0.254	η_p_^2^ = 0.023
		Treatment	F_1,57_ = 0.010	P = 0.920	η_p_^2^<0.001
		Rearing x Sex	F_1,57_ = 0.509	P = 0.479	η_p_^2^ = 0.009
		Rearing x Treatment	F_1,57_ = 0.657	P = 0.421	η_p_^2^ = 0.011
		Sex x Treatment	F_1,57_ = 1.422	P = 0.238	η_p_^2^ = 0.024
		Rearing x Sex x Treatment	F_1,57_ = 0.268	P = 0.607	η_p_^2^ = 0.005
	Doubles Index	Rearing	F_1,57_ = 2.908	P = 0.094	η_p_^2^ = 0.049
		Sex	F_1,57_ = 0.243	P = 0.624	η_p_^2^ = 0.004
		Treatment	F_1,57_ = 15.59	P = 0.0002	η_p_^2^ = 0.215
		Rearing x Sex	F_1,57_ = 3.346	P = 0.073	η_p_^2^ = 0.055
		Rearing x Treatment	F_1,57_ = 7.716	P = 0.007	η_p_^2^ = 0.119
		Sex x Treatment	F_1,57_ = 6.265	P = 0.015	η_p_^2^ = 0.099
		Rearing x Sex x Treatment	F_1,57_ = 0.166	P = 0.685	η_p_^2^ = 0.003
	**Two-Way ANOVA**				
		**Comparison**	**Test Statistic**	**P**	**η**_**p**_^**2**^
	Female Doubles Index	Rearing	F_1,28_ = 5.445	P = 0.027	η_p_^2^ = 0.162
		Treatment	F_1,28_ = 18.14	P = 0.0002	η_p_^2^ = 0.393
		Rearing x Treatment	F_1,28_ = 4.422	P = 0.045	η_p_^2^ = 0.136
	Male Doubles Index	Rearing	F_1,29_ = 0.009	P = 0.926	η_p_^2^<0.001
		Treatment	F_1,29_ = 1.213	P = 0.280	η_p_^2^ = 0.040
		Rearing x Treatment	F_1,29_ = 3.26	P = 0.081	η_p_^2^ = 0.101
	**Relevant Post Hoc Tests**				
		**Comparison**	**Test Statistic**	**P**	**Cohen’s d**
	Female Doubles Index	EL: MS vs Con	T_28_ = 3.241	P = 0.0092	d = 1.261
		CL: MS vs Con	T_28_ = 0.158	P = 0.876	d = 0.137
		MS: EL vs CL	T_28_ = 4.516	P = 0.0004	d = 2.215
		Con: EL vs CL	T_28_ = 1.519	P = 0.261	d = 0.781
**P10 Cohort**	**Three-Way ANOVA**				
		**Comparison**	**Test Statistic**	**P**	**η**_**p**_^**2**^
	Arm Entries	Rearing	F_1,58_ = 0.020	P = 0.889	η_p_^2^<0.001
		Sex	F_1,58_ = 6.127	P = 0.016	η_p_^2^ = 0.100
		Treatment	F_1,58_ = 1.003	P = 0.321	η_p_^2^ = 0.017
		Rearing x Sex	F_1,58_ = 3.785	P = 0.057	η_p_^2^ = 0.061
		Rearing x Treatment	F_1,58_ = 0.025	P = 0.876	η_p_^2^<0.001
		Sex x Treatment	F_1,58_ = 1.441	P = 0.235	η_p_^2^ = 0.024
		Rearing x Sex x Treatment	F_1,58_ = 0.308	P = 0.581	η_p_^2^ = 0.005
	Alternation Index	Rearing	F_1,58_ = 0.056	P = 0.814	η_p_^2^<0.001
		Sex	F_1,58_ = 0.090	P = 0.765	η_p_^2^ = 0.002
		Treatment	F_1,58_ = 3.374	P = 0.071	η_p_^2^ = 0.060
		Rearing x Sex	F_1,58_ = 0.295	P = 0.589	η_p_^2^ = 0.005
		Rearing x Treatment	F_1,58_ = 2.086	P = 0.154	η_p_^2^ = 0.035
		Sex x Treatment	F_1,58_ = 4.049	P = 0.049	η_p_^2^ = 0.070
		Rearing x Sex x Treatment	F_1,58_ = 0.014	P = 0.905	η_p_^2^<0.001
	Doubles Index	Rearing	F_1,58_ = 0.278	P = 0.600	η_p_^2^ = 0.005
		Sex	F_1,58_ = 0.160	P = 0.691	η_p_^2^ = 0.003
		Treatment	F_1,58_ = 0.098	P = 0.755	η_p_^2^ = 0.002
		Rearing x Sex	F_1,58_ = 2.793	P = 0.100	η_p_^2^ = 0.046
		Rearing x Treatment	F_1,58_ = 0.353	P = 0.555	η_p_^2^ = 0.006
		Sex x Treatment	F_1,58_ = 0.485	P = 0.489	η_p_^2^ = 0.008
		Rearing x Sex x Treatment	F_1,58_ = 0.816	P = 0.370	η_p_^2^ = 0.014
	**Two-Way ANOVA**				
		**Comparison**	**Test Statistic**	**P**	**η**_**p**_^**2**^
	Female Alternation Index	Rearing	F_1,30_ = 0.073	P = 0.788	η_p_^2^ = 0.002
		Treatment	F_1,30_ = 11.54	P = 0.002	η_p_^2^ = 0.277
		Rearing x Treatment	F_1,30_ = 1.366	P = 0.252	η_p_^2^ = 0.044
	Male Alternation Index	Rearing	F_1,28_ = 0.217	P = 0.645	η_p_^2^ = 0.007
		Treatment	F_1,28_ = 0.011	P = 0.917	η_p_^2^<0.001
		Rearing x Treatment	F_1,28_ = 0.872	P = 0.358	η_p_^2^ = 0.030

Statistical results for Experiment 2: Spontaneous alternation behavior in adulthood. Significant findings are shaded gray. Empty liposomes (EL), clodronate liposomes (CL).

### 3.3 Microglial morphology

It is intriguing that the P10-treated empty liposome cohort did not show the same MS-driven impairment in working memory as our P2-treated empty liposome cohort (Fig 2D and 2H), given that empty liposome treatment was meant to serve as a benign control treatment. However, it is possible that empty liposome treatment has an effect on PFC microglia depending on the age of injection. Therefore, in order to test if microglial morphology changed following empty liposome treatment in an age-dependent manner, pups were given saline or empty liposome i.c.v. injections on P2 or P10, and their frontal cortices stained for the microglial marker Iba1 for morphological analysis (Fig 3A–3D). 2-way ANOVAs revealed main effects of age on microglial branchpoints (F_1,18_ = 667.8, P<0.0001, η_p_^2^ = 0.908) (Fig 3E), ramification index (F_1,18_ = 462.7, P<0.0001, η_p_^2^ = 0.963) (Fig 3F), percent of microglial occupied territory (F_1,18_ = 1353, P<0.0001, η_p_^2^ = 0.251) (Fig 3G), and soma area (F_1,18_ = 58.81, P<0.0001, η_p_^2^ = 0.766) (Fig 3H). There were no main effects of treatment on microglial branchpoints, ramification index, or percent occupied space. While there were no significant main effects of treatment or interactions on any of these morphological categories, it should be noted that the effect of treatment on soma area had a large effect size (F_1,18_ = 3.496, p = 0.0799, η_p_^2^ = 0.163), though this did not reach significance. Together these results indicate that microglia shift toward being more ramified with smaller soma between P3 and P11.

A 2-way ANOVA revealed main effects of age (F_1,18_ = 148.7, P<0.0001, η_p_^2^ = 0.892) and treatment (F_1,18_ = 7.117, P = 0.0157, η_p_^2^ = 0.283; Fig 3I) on the percent of amoeboid microglia but also an age by treatment interaction (F_1,18_ = 6.0, P = 0.0245, η_p_^2^ = 0.251). Holm-Sidak’s multiple comparisons test showed a significant difference between saline and empty liposome-treated animals on P2 (t_18_ = 3.842, P = 0.0024, d_Cohen_ = 1.664) but not on P10 (t_18_ = 1431, P = 0.8878). Additionally, there were significant differences between both saline-treated animals on P2 compared to P10 (t_18_ = 9.827, P<0.0001, d_Cohen_ = 6.384) and empty liposome-treated animals on P2 compared to P10 (t_18_ = 7.306, P<0.0001, d_Cohen_ = 4.197), highlighting the main effect of age on percent of amoeboid microglia (Fig 3I). For full statistical results see Table 3. These results indicate that empty liposomes decreased the percent of amoeboid microglia when administered on P2, but not when administered on P10.

**Table 3:** Microglial morphology statistical results.

	**Two-Way ANOVA**			
	**Comparison**	**Test Statistic**	**P**	**η**_**p**_^**2**^
**Branchpoints**	Treatment	F_1,18_ = 1.001	P = 0.330	η_p_^2^ = 0.053
	Age	F_1,18_ = 177.6	P<0.0001	η_p_^2^ = 0.908
	Age x Treatment	F_1,18_ = 0.257	P = 0.6187	η_p_^2^ = 0.014
**Ramification Index**	Treatment	F_1,18_ = 0.509	P = 0.485	η_p_^2^ = 0.028
	Age	F_1,18_ = 462.7	P<0.0001	η_p_^2^ = 0.963
	Age x Treatment	F_1,18_ = 0.548	P = 0.469	η_p_^2^ = 0.030
**Percent Occupied Area**	Treatment	F_1,18_ = 0.763	P = 0.394	η_p_^2^ = 0.041
	Age	F_1,18_ = 1353	P<0.0001	η_p_^2^ = 0.987
	Age x Treatment	F_1,18_ = 0.674	P = 0.422	η_p_^2^ = 0.036
**Soma Area**	Treatment	F_1,18_ = 3.496	P = 0.0779	η_p_^2^ = 0.163
	Age	F_1,18_ = 58.81	P<0.0001	η_p_^2^ = 0.766
	Age x Treatment	F_1,18_ = 0.806	P = 0.3811	η_p_^2^ = 0.043
**Percent Amoeboid**	Treatment	F_1,18_ = 7.117	P = 0.016	η_p_^2^ = 0.283
	Age	F_1,18_ = 148.7	P<0.0001	η_p_^2^ = 0.892
	Age x Treatment	F_1,18_ = 6.024	P = 0.025	η_p_^2^ = 0.251
	**Relevant Post Hoc Tests**			
	**Comparison**	**Test Statistic**	**P**	**Cohen’s d**
**Percent Amoeboid**	P2: Sal vs EL	T_18_ = 3.842	P = 0.0024	d = 1.664
	P10: Sal vs EL	T_18_ = 0.1431	P = 0.8878	d = 0.541
	Sal: P2 vs P10	T_18_ = 9.827	P<0.0001	d = 6.384
	EL: P2 vs P10	T_18_ = 3.842	P = 0.0024	d = 1.664

Statistical results for Experiment 3: Microglial morphology analysis. Significant findings are shaded gray. Saline (Sal), empty liposomes (EL).

## 4. Discussion

This study initially sought to test whether microglia mediate the long-term impacts of ELA on working memory, as assessed by the spontaneous alternation task. We hypothesized that rats exposed to MS would display impaired working memory as a result of ELA-induced changes in microglia, such that if their microglia were depleted during MS, these impairments would be prevented. As microglia are difficult to manipulate experimentally, the pharmacologic agent clodronate liposomes were used to transiently deplete microglia, with empty liposomes used as a control. Given that microglia undergo rapid development during the pre-weaning period, microglia were depleted at two different timepoints to assess if there were specific developmental windows when microglial activity mediates the effects of ELA.

This study aimed to investigate microglial depletion both early and late during the MS paradigm. P2 was chosen as the early timepoint to coincide with the first day of MS. Microglia repopulate the brain approximately eight days following P2 clodronate liposome treatment. Clodronate liposome-treated animals displayed reduced microglia count four and six, but not eight, days following the infusion compared to empty liposome-treated animals. Therefore, P10 was chosen to assess if microglia mediate the effects of MS during a later window in the paradigm. Clodronate liposome treatment on P10 similarly depleted microglia for eight days following the infusion. Other studies using clodronate liposomes in rat pups showed transient depletion of microglia over the first postnatal week, however the specific patterns of repopulation may be influenced by the dosage of clodronate liposomes and brain area investigated [54,57].

During the postnatal period microglia undergo many changes, including increases in cell number, increases in ramification, and shifting functions to accommodate brain maturation [28,62–65]. In the present study, we found a developmental increase in the microglial cell count of empty liposome-treated animals between P6 and P8 that remains through P10, and others have shown that microglia count peaks at P14 [63,64]. This developmental trajectory of microglial cell number should be considered when comparing the depletion at different timepoints. Though clodronate liposome treatment led to an eight-day reduction in microglia in both conditions, the level of depletion may not be the same in each condition. Four days after the early depletion, the average microglia count in empty liposome treated animals was approximately 2,318 microglia higher than the clodronate treated animals (d_Cohen_ = 2.577 see Table 1), likewise four days after the late depletion the empty liposome treated animals was approximately 2,455 microglia higher than in the clodronate treated animals (d_Cohen_ = 2.984 see Table 1). While the estimated absolute number of microglia that are depleted are similar between the two different time points, because there are fewer microglia in the brain on P2 than on P10 the relative reductions are not equal. Therefore, the same dose of clodronate liposomes may not reduce microglia count to the same extent at two different timepoints. Moreover, based on the developmental neonatal atlas used in this study [58], the brain, excluding the olfactory bulbs and cerebellum, grows approximately 3.4 mm between P2 and P10. Therefore, in the P10 microglia manipulated animals, the liposomes have further to travel in order to reach the frontal cortex, and as a result may have a greater impact on circumventricular regions. This is an important consideration, as other regions, such as the hippocampus, are important for working memory [59,66]. Future studies could assess local microglial manipulations to the PFC or hippocampus to assess region specific impacts of microglia on cognitive function.

Another aspect of this depletion method to consider is how the microglia that survive depletion and the microglia that repopulate the brain are different from unadulterated microglia. There are various proposed mechanisms by which microglia repopulate the brain following depletion. While one study indicated that microglia are repopulated from nestin+ progenitor cells following CSF1R inhibition [50], another study using genetic ablation showed that microglia are repopulated with local proliferation of the remaining pool of microglia [51]. A more recent study also found that following CSF1R inhibition, microglial repopulation occurs from the remaining microglia; there was no evidence for repopulation from progenitor cells nor from blood cells [52]. Moreover, one study using liposomal clodronate in juvenile rats showed that repopulated Iba1+ cells were also P2YR12+, indicating these cells were not peripheral immune cells [67]. Because genetic approaches, CSF1R inhibition, and juvenile clodronate liposomes result in microglial repopulation from the existing pool of microglia, it is plausible the same mechanism occurs following clodronate liposome treatment in neonates; however, this hypothesis has not been tested directly. Additionally, it is unclear how these repopulated microglia may differ from unadulterated microglia, and the impacts this may have on development of the microglia themselves, neural circuits, and subsequent behavior.

Previous literature has demonstrated that ELA negatively affects cognition [46,47,68]. Here, we used the spontaneous alternation test to assess working memory, which is one domain of cognition entailing the ability to retain information for a short time and use it in the execution of a task. Many mammals and nonmammals with intact working memory exhibit spontaneous alternation behavior (SAB), which is the tendency to enter each arm of a maze in succession. Performing doubles, when an animal enters the same arm of a maze twice in a row, is a sign of impaired working memory [69]. Because MS yields later-life neuroinflammatory markers such as increased proinflammatory cytokines [45] and increased microglia soma size [44] in regions important for working memory, such as the PFC, we tested whether depleting microglia during MS prevented impairments in working memory. We found that the effect of MS on both the number of arm entries and the doubles index depended on the age at which the microglial manipulation occurred. In the cohort that received early postnatal (P2) infusions, MS animals made fewer total arm entries. This is unsurprising as previous work from our lab has shown an MS-induced decrease in locomotion in the elevated zero maze, although this effect was previously seen in females only [70]. In the cohort that received later infancy (P10) infusions, there was a significant main effect of sex such that females made fewer arm entries. Of note, doubles and alternations may be differentially influenced by general locomotion. Thus, we calculated a doubles and alternation index for all animals, rather than comparing the raw number of doubles and alternations, to obviate any bias due to differences in arm entries alone. MS impaired working memory in P2 empty liposome treated females, as indicated by a higher doubles index compared to control reared females that received empty liposomes; however, this impairment was prevented by clodronate liposome administration. Therefore, in females, the presence of microglia, which had been treated with empty liposomes, during MS from P2-P10 yielded a deficit in adult working memory. If liposomes contained clodronate, rather than being empty, microglia were depleted, which precluded MS and the liposome itself from provoking the change in microglial function that led to the increase in the doubles index. However, the mechanisms by which these microglia lead to deficits in working memory is unknown. For example, the developmental microglial refinement of working memory circuits could be altered or there could be long-term changes in microglia that negatively affect their activity during adult working memory tasks.

Given the results of the first cohort, we hypothesized that if the second half of MS also impacts microglial function for long-term impacts on working memory, then depleting microglia on P10 would also prevent the MS-induced increase in the doubles index in females. On the other hand, if the second half of MS does not influence microglial function, then depleting microglia on P10 would not prevent the MS-induced impairment in working memory. Surprisingly, however, MS did not impair working memory in the empty liposome groups within the P10-treated cohort, failing to replicate the MS effects on working memory observed in the P2 cohort. While these results were unexpected, the current literature surrounding the effect of ELA on SAB is limited. Studies demonstrating reduced SAB following MS have mostly been focused on the adolescent timepoint [71,72], and there are few studies investigating effects of ELA on SAB in adulthood. One study showed that mice exposed to limited bedding and nesting had fewer alternations in adulthood [73], and another study found that mice exposed to MS showed no difference in alternations compared to controls [74]. We theorized, therefore, that MS-induced changes to SAB do not typically persist into adulthood, but that the methods used in the present study influenced the effect of MS in the P2 cohort.

Although there were no impacts of MS on working memory in the later-treated cohort, microglial depletion at P10 impaired adult working memory. Females treated with clodronate liposomes on P10 performed fewer alternations compared to females treated with empty liposomes, regardless of rearing condition. This finding highlights the importance of microglia in the development of cognition related neural circuits, especially as they regulate the formation and maintenance of synapses. For example, neonatal microglial depletion in mice reportedly results in supernumerary parvalbumin inhibitory synapses. This increase in synapses persisted into adolescence [75], which is a time when the PFC undergoes reorganization to support cognitive maturation [76]. Therefore, depleting microglia from P10 to P18 may prevent microglial interactions with synapses during development, which could impact the cortex during an important window for the scaffolding of neural circuits involved in cognition. However, it is interesting that this impairment was seen in females specifically. There are limited studies in sex-differences in synapse refinement in the PFC, however one study demonstrated that bouton number peaked in females at P35 in the PFC, which was not seen in males [77]. Therefore, microglial depletion during the second week of life may differentially impact male and female developmental trajectories. The impacts of depletion on maintenance of cognitive circuits may depend on the sex-specific brain architecture at the time of depletion and may also alter the developmental trajectory of these circuits.

While the MS paradigms were identical between the animals receiving P2 and P10 injections, the protocols for anesthesia differed based on injection timepoint. Pups injected on P2 underwent 10–12 min of cryoanesthesia while pups injected on P10 underwent 10–12 min of isoflurane inhalation anesthesia. There is some evidence to suggest that neonatal cold exposure can influence cognition and cognition related brain regions. Adult Long-Evans rats exposed to 60 minutes of neonatal hypothermia reportedly had a longer latency and pathlength in the Morris Water Maze compared to adults who had not been exposed to cold. Additionally, 15 minutes of neonatal cold exposure decreased the volume of the dentate gyrus in the adulthood compared to animals who had not been exposed to the cold [78]. Other studies using cold exposure as a stressor have shown changes in microglial morphology [40] and an increase in pro-inflammatory cytokines in the hippocampus [79]. Stress can prime microglia to be more reactive to later life challenges; therefore, it is possible that cold exposure early in the postnatal period primes microglia to be more reactive to a second stressor, in this case MS. Thus, one possible explanation for the observed cognitive effects of MS in the P2 injected animals is that the cognitive effects are a result of the combination of cold exposure in addition to MS. However, this hypothesis would need to be tested directly.

Maternal care is also an important facet of pup development [80]. While maternal care was not measured directly in this study, we have previously reported that MS dams display fragmented care an hour and a half following pup reunion on P9 [81]. However, it is unknown if the dams here bestowed differential care towards pups treated with empty liposomes versus clodronate liposomes. Moreover, the timing of the manipulation could play a role in maternal care differences as maternal care shifts throughout the neonatal period [82]. Therefore, it is unclear if or how maternal care is altered based on the interaction of rearing, microglial presence, and the timing of microglial manipulation and if this played any role in the long-term behavioral effects reported in the present study.

Another possible explanation for the MS-driven cognitive impairments seen in the P2 injected rats but not the P10 injected rats is that the liposome treatment itself affects microglial morphology and function. While the empty liposomes are a control for clodronate liposomes, microglia are still undergoing phagocytosis that may not have been occurring during that developmental time window or at that rate. Notably, empty liposomes themselves can change cytokine release and baseline phagocytic activity. This phagocytosis can alter microglia transcriptionally and translationally [83,84]. As microglia continue to develop in the postnatal period, and are subject to early life programming, we sought to determine whether empty liposome treatment altered microglia morphology in an age-dependent manner [65,85,86]. We found that empty liposome treatment decreased the percent of amoeboid microglia following P2 treatment but not P10 treatment. These findings provoke more questions about the impact that liposome treatment has on microglial development, and how this may impact the building and maintenance of neural circuits. For example, microglia become more ramified over development, seen clearly by the main effect of age on percent of amoeboid microglia. Thus, it may be possible that liposomes accelerate this development. Future studies could investigate the duration that liposome treatment changes microglial morphology following administration. Another question is whether these morphological changes in microglia are paired with functional changes in microglia. For example, how does liposome treatment early in development change the transcriptional profile of microglia, cytokine release, or phagocytic capabilities both in the short and long term? Given the data from the morphological analyses we hypothesize that altering microglia with liposomes on P2 combined with exposure to MS additively impair working memory in adulthood. Future work will be needed to directly test this hypothesis.

## 5. Conclusions

The present study investigated the role that microglia play in the long-term impacts of ELA on working memory. Using clodronate liposomes to transiently deplete microglia at two different timepoints during a maternal separation paradigm, we found a cohort specific effect. Female rats who received empty liposomes on P2 and were exposed to MS displayed impairments in the spontaneous alternation task. However, if MS-exposed female rats had their microglia depleted from P2-10, these impairments were prevented. In a separate cohort, rats who received empty liposomes on P10 and were exposed to MS did not show any impairments in the spontaneous alternation task, but microglial depletion from P10-P18 impaired working memory in females. One potential explanation for this discrepancy is that empty liposome treatment on P2, but not P10, changed microglial morphology 24 hours after the infusion. Developmental manipulations of microglia can have long lasting impacts on their function, which may influence the effect of additional stressors. These findings suggest that throughout the female pre-weaning period, microglia are important for later-life working memory. Specifically, MS compounded with an additional challenge in the early pre-weaning period alters their function resulting in impaired working memory, whereas in the late pre-weaning period microglial presence supports intact working memory, regardless of environmental challenge. Future studies are necessary to understand the impacts of both liposome treatment and maternal separation on microglial function and how these cascades influence working memory throughout the lifespan in a sex-dependent manner.

## Supporting information

S1 Data Raw data for Experiments 1, 2, and 3. (XLSX)
